# Environmental impacts of forest road construction on mountainous terrain

**DOI:** 10.1186/1735-2746-10-23

**Published:** 2013-03-15

**Authors:** Erhan Caliskan

**Affiliations:** 1 Faculty of Forestry, Karadeniz Technical University, 61080, Trabzon, Turkey

**Keywords:** Environmental impacts, Forest road construction, Buldozer, Excavator, Mountainous terrain

## Abstract

Forest roads are the base infrastructure foundation of forestry operations. These roads entail a complex engineering effort because they can cause substantial environmental damage to forests and include a high-cost construction. This study was carried out in four sample sites of Giresun, Trabzon(2) and Artvin Forest Directorate, which is in the Black Sea region of Turkey. The areas have both steep terrain (30-50% gradient) and very steep terrain (51-80% gradient). Bulldozers and hydraulic excavators were determined to be the main machines for forest road construction, causing environmental damage and cross sections in mountainous areas.

As a result of this study, the percent damage to forests was determined as follows: on steep terrain, 21% of trees were damaged by excavators and 33% of trees were damaged by bulldozers during forest road construction, and on very steep terrain, 27% of trees were damaged by excavators and 44% of trees were damaged by bulldozers during forest road construction. It was also determined that on steep terrain, when excavators were used, 12.23% less forest area was destroyed compared with when bulldozers were used and 16.13% less area was destroyed by excavators on very steep terrain. In order to reduce the environmental damage on the forest ecosystem, especially in steep terrains, hydraulic excavators should replace bulldozers in forest road construction activities.

## Introduction

Forest roads are the most costly structures in forestry. Inadequately constructed forest roads can cause severe environmental impacts including road surface erosion and sediment yield [1,2], pollution of off-site waters [3-5], slope failures and mass movement [6,7] direct loss of habitat (by the conversion of the original land cover into an artificial surface) [8] and indirect loss of habitat (by the fragmentation of an ecosystem into smaller and more isolated patches) [9-11]. Therefore, forest road managers should design forest roads by considering not only cost efficiency but also sustainable management of the forest environment [12,13]. During the construction project of a forest road, the standard design must be carried out on the ground to achieve the desired road with minimal impact on environment [14]. Sometimes the standard design cannot be useful for determining the clearing limit of forest roads [15].

Large areas of forest are destroyed during road construction which not only results in economic losses, but also changes the conditions of the environment [16]. Forest road construction is a hazardous operation in mountainous terrain and can inflict scars on the landscape and also cause substantial damage to the forest ecosystem.

One of the negative effects of road construction is the loss of forest area. The ecological balance in forests is adversaly affected by rockfall and forest road costruction work [17,18]. The proliferation of human-made clearings may have important impacts on wildlife populations [19]. The clearance of a forest road cross-section affects both the forest and the road [20]. One of the first steps in forest road construction is the clearing of trees. At this phase, trees and other large vegetation within the construction boundaries are cut down. In addition, hazardous roots and unsafe trees adjacent to the area should also be cut down [21].

Forest road construction often results in the most environmental impact to adjacent ecosystems because earth movement and other activities can disturb whole watersheds [22]. Heinrich [23] indicated that excavators have been commonly used in environmentally sensitive areas to reduce impact on forest vegetation, provide adequate drainage systems, protect stream crossings, and improve stabilization of cut and fill slopes.

Forest roads are built through the excavation of soil and rock. Rockfall occurs during construction and is caused by excavated rock pieces on embankment slopes and the blasting of block rock masses. In Turkey, the traditional use of bulldozers causes loss of land and damage to trees and forest habitat.

In this study, forest road construction techniques using hydraulic excavators and bulldozers were investigated based on sample road construction activities conducted on forest lands in Giresun, Trabzon and Artvin in Turkey. The environmental damage, cross sections, and use of hydraulic excavators and bulldozers were evaluated. The results of this research will be useful in regulating forest road construction projects and monitoring the use of bulldozers and hydraulic excavators.

## Material and methods

### Study areas

**Figure 1:**
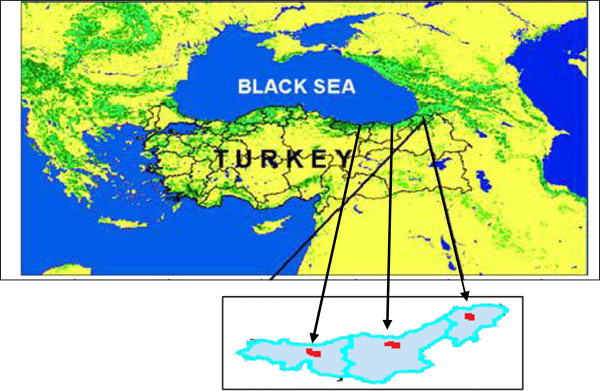
The study area.

Four areas in which forest road construction work is done and which have similar characteristics were within the Giresun, Trabzon and Artvin mountain areas, chosen for the area of study (Figure 1). In the Şalpazarı Forest Enterprise area (Trabzon), commercial tree species include *Picea orientalis*. The elevation ranges from 700 to 800 meters with ground slopes of 30 to 50%. Total length of the sample road examined in this study was about 1,550 meters with an average road width of 4 meters. In the Düzköy Forest Enterprise area (Trabzon), commercial tree species include *Fagus orientalis* and *Picea orientalis*. The elevation ranges from 800 to 900 meters with ground slopes of 30 to 50%. Total length of this sample road was about 1,540 meters and the average road width was 4 meters. In the Kulakkaya Forest Enterprise area (Giresun), commercial tree species include *Fagus orientalis* and *Picea orientalis*. The elevation ranges from 1,200 to 1,300 meters with ground slopes of 51 to 80%. Total length of this sample road was about1,500 meters and the average road width was 4 meters. In the Madenler Forest Enterprise area (Artvin), commercial tree species include *Picea orientalis*. The elevation ranges from 1,100 to 1,350 meters with ground slopes of 51 to 80%. Total length of this sample road was about 1,500 meters and the average road width was 4 meters. The soil type of each of the areas was similar (rocky soil).

During construction, CAT D7 and D9 bulldozers and CAT 330 and Komutsu PC220 hydraulic excavators were used. Also, in order to break up the rocks, one rear-mounted tooth ripperon a bulldozer was used. The excavators were equipped with hydraulic hammers instead of the metallic buckets.

### Field study

**Figure 2:**
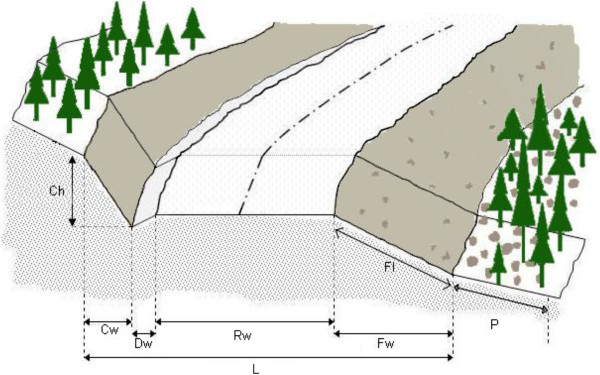
The decision variables measured from each cross section along the sample road.

The entire construction process in the areas of the study was observed in the field, and data was collected during and after construction. To characterize road sections, nine decision variables were measured from each cross section. These variables include cut-slope height (Ch), cut-slope width (Cw), ditch width (Dw), road width (Rw), fill-slope width (Fw), fill-slope length (Fl), road construction zone width (L), effect distance length (P), and ground slope (S) (Figure 2). Surveying instruments such as clinometers, steel tape, laser range-finders, miras, poles and altimeters were used in the field studies.

In the forest roads constructed, averaging 1,500 meters in total, 30 test fields each with a width of 10 meters were chosen every 50 meters. The place of the test fields in which samples would be taken by systematic sampling was determined. Two cross-sections were taken at the beginning and at the end of each test field and 60 measurements were completed. It was found that most of the damage to the trees below the roads was caused by machines in the first ten meters. For each study area, the shapes of the damage under constructed roads were investigated. The types of damage such as bending of trees, crushing of trees and damage to roots were observed. Distribution by damage type related to construction tecniques and the effects of positional values of damaged trees were investiged by means of the data collected.

### Statistical analyses

The average and standard error values obtained from cross-sections were calculated. Necessary graphics were drawn using the SPSS statistical package program. The number of damaged trees (Dt) was considered a dependent variable and gradient of the fill slope (Fs), cut area (Ca), slope of the terrain(Ts), length of the fill slope (Fl), effect distance length (P), width of the roadway (Rw), number of the trees (Tn), type of the machine (Mt) such as excavator=1, bulldozer=2, and the road gradient (Gr) were considered independent variables and correlation analysis was carried out. Then, in order to find the most effective independent variables on the number of the damaged trees, regression analysis was made with the help of the SPPS package program.

## Results and discussion

Average values belonging to the measurements collected at the cross-sections which were at the test fields of all the research areas are given in Table 1.

**Table 1:** The values of decision variables measured on the cross sections

**Variables**	**Symbol**	**Excavator I**	**Bulldozer I**	**Excavator II**	**Bulldozer II**
Cut-slope height (m)	Ch	2,45	3,64	4,93	5,26
Cut-slope width (m)	Cw	1,15	1,18	1,34	1,35
Ditch width (m)	Dw	1,05	1,05	1,05	0,98
Road width (m)	Rw	4,12	4,21	4,25	4,19
Fill-slope width (m)	Fw	4,49	6,33	5,19	9,77
Fill-slope lenght (m)	Fl	3,60	4,89	3,16	3,76
Gradient of the terrain (%)	Tg	48,87	45,57	68,83	59,83
Effect distance (m)	P	2,78	5,10	3,82	5,32
Width of the construction area (m)	L	10,06	10,91	11,62	13,32
Cut area (m ^2^)	Ca	1,51	2,34	3,31	3,54

From Table 1, it can be seen that when the bulldozers were used for road construction in both slope groups, the values measured at the road cross-sections such as the height of the cut slope, width of the cut slope, length of the fill slope, width of the fill slope, the length of the area affected by road construction, width of the construction area and cut area increased.

It was observed that the width of the construction area was smaller when excavators were used. For that reason, less forest area was destroyed and fewer trees were cut of in order to clear the way for the road.

In this study, the average construction zone width in ExcavatorI, BulldozerI, Bulldozer II, and Excavator II were found to be 10.06 meters, 10.91 meters, 11.62 meters and 13.32 meters, respectively. A study conducted in the Antalya region [15] reported that road construction on a terrain with 36 - 50% ground slope resulted in 9.40 and 12.18 meter wide road construction zones using excavators and bulldozers, respectively. This suggested that the impacted forested area using bulldozers was approximately 29.58% greater than that of using excavators. Öztürk and İnan [24] also revealed in the Aykiricay region that the average construction zone width was 6.22 meters, and therefore the sample road section impacted approximately 1.00 ha of the forested area (6.22 x 1,650 meters road length) during road construction. In the Seyitgazi region, average zone width was 7.47 meters and the sample road section impacted 2.24 ha of the forested area (7.47 x 3,000 meters). The differences in cut-slope and fillslope areas between excavators and bulldozers were measured.

**Figure 3:**
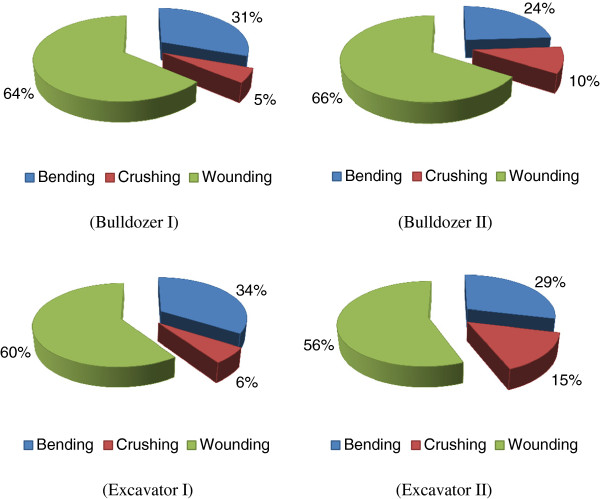
The percent of damage as bending, crushing and wounding.

The percent of damage in terms of wounding, bending and crushing of trees are 64%, 31% and 5% repectively (for BulldozerI), 66%, 24% and 10% repectively (for BulldozerII), 60%, 34% and 6% repectively (for ExcavatorI) and 56%, 29% and 15% repectively (for Excavator II). The percent of damage in terms of wounding, bending and crushing is shown in Figure 3. The number and rate of the damaged trees in the study areas are shown in Table 2.

**Table 2:** Number and rate of damaged trees in study areas

**Machine type**	**Average terrain slope(%)**	**Number of damage trees**	**Number of non damage trees**	**Types of damages**			**Number of total trees**	**Damage rate (%)**
				**Bending**	**Crushing**	**Wounding**		
Excavator I	48	47	175	16	3	28	222	21
Bulldozer I	45	72	144	22	4	46	216	33
ExcavatorII	68	66	170	19	10	37	236	27
BulldozerII	59	100	129	24	10	66	229	44

The percent of damage rate for Excavator I, Excavator II, Bulldozer I and Bulldozer II are 21%, 27%, 33% and 44% repectively.

In this study, damage caused by excavators is less than that caused by bulldozers. Previous studies have also indicated this. Furthermore, forest roads constructed using hydraulic excavators are much more visually appealing than those using bulldozers, in terms of both technical and environmental aspects [25].

**Figure 4:**
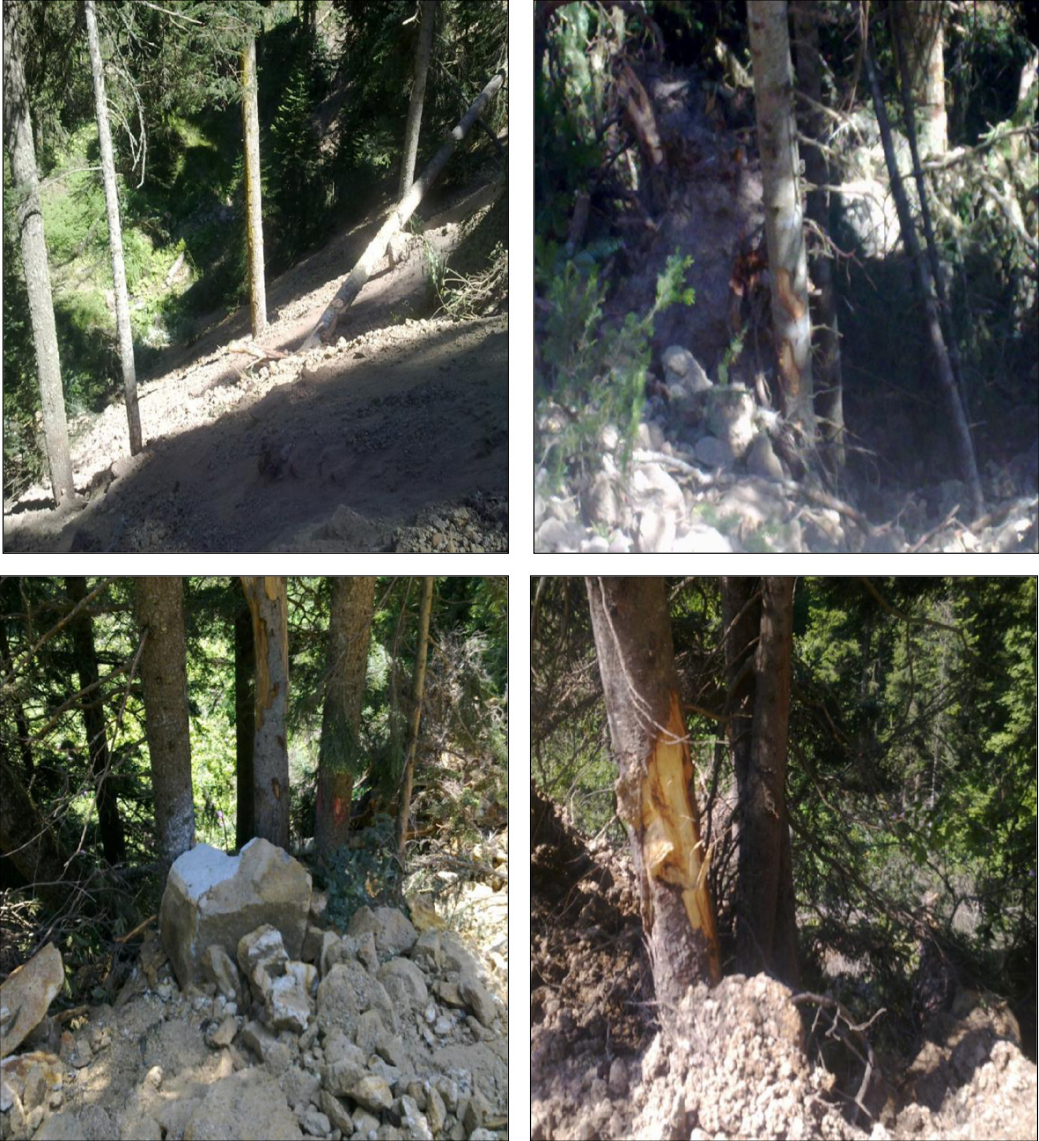
Tree damages during forest road construction.

In each study area, bark beetles may damage trees after road construction. These bark beetles caused major damage in these regions. Also, direct economic loss occurred due to poor timber quality. The damage caused by road costruction are shown in Figure 4.

In a study by Tochiki and Kaibori [26], it was stated that the major cause of damage on slopes and the sliding of materials along forest roads in the northwest region of Hiroshima was the water that ran along the road after heavy rain.

In another study, it is stated that bark insects can easily cause epidemics on the trees which are damaged by stones and rocks, and that fungi and other harmful organisms destroy 50% of first class timber trees [27].

**Figure 5:**
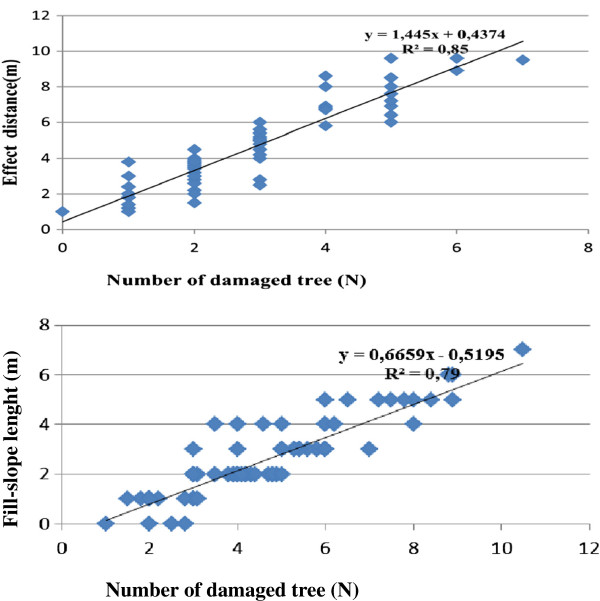
Relationship between the number of damaged trees, the length of effect distance and the length of fill slope.

A correlation analysis was performed on the dependent variable of the number of damaged trees and the other nine independent variables. As a result of the correlation analysis, it was determined that there was a significant relationship at a 99% confidence level between the number of damaged trees and independent variables such as gradient of the terrain, cut area, length of the fill slope, length of the effect distance, number of trees and type of machine. The graphics of the relationships that are considered to be important in the correlation matrix are given below. In the graphics, it can be seen that as the length of effect distance increases, the number of damaged trees also increases. In the same way, as the length of the fill slope increases, the number of damaged trees again increases (Figure 5).

At the construction sites, the number of damaged trees (Dt) was taken as the dependent variable and the other nine variables as independent. Then, a regression analysis was conducted. The equation of the fitted model is as follows:

$$\begin{array}{l} \mathrm{Dt}=-0,448+0,06\times \mathrm{Ts}+0,310\times \mathrm{Fl}-0,025\\ \;\times \mathrm{Fw}-0,011\mathrm{Fs}+0,370P+0,021\mathrm{AS}\\ \;+0,085\mathrm{MT},\left(R^{2}\right)\\ \;=0,92 \end{array}$$

The *R*^*2*^ of 0.92 indicates that 92% of the variations of dependent variables can be explained by the model. As a result of the multiple linear regression analysis, it was observed that the most important independent variables that had an effect on the number of damaged trees were slope terrain, length of the fill slope, width of the fill slope, slope of the fill slope effect distance, the number of the trees and type of machine.

**Figure 6:**
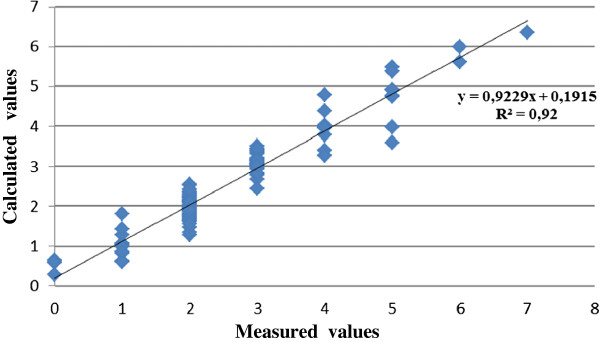
A comparison between the calculated and measured values of number of damaged trees.

It was concluded that the number of damaged trees increased as the values of the number of the trees in the construction sites, length of the fill slope and effect distance increased and when bulldozers were used instead of excavators. The graphics of the measured values and the calculated values belonging to the regression model are seen in Figure 6.

It was found that 1.7 hectares of forest were lost when a bulldozer was used and 1.5 hectares were lost when an excavator was used on steep terrain (30-50% slope), for a 1.5 kilometer-long road. If in very steep terrain (51-80% slope), for the same length of road, 2.05 hectareswere lost when a bulldozer was used and 1.74 hectares were lost when an excavator was used. It is clear that the largest forest area was destroyed in very steep terrain where the bulldozer was used. However, in steep terrain where an excavator was used, it was observed that the least amount of forest was destroyed. When an excavator was used in very steep terrain, it destroyed nearly the same amount of forest as a bulldozer would in steep terrain.

## Conclusion

In this study, the forest road construction techniques using both hydraulic excavators and bulldozers were evaluated considering environmental issues. The following are recommended findings:

1. For protecting the natural environment, using well-equipped and powerful excavators must be given preference overusing bulldozers, especially for determining the path and limits of the working area in steep terrains.

2. In mountainous areas, clearing of trees should be kept to a minimum to prevent soil erosion. When working close to waterways, it may be necessary to take precautions to prevent sediment from washing into streams. Preventative measure may include installation of silt traps or silt screens.

3. The most important role the equiment operator plays during road construction is to determine the safest and most efficent use of equipment.

4. Forest road managers should consider not only the total road cost but also enviromental impact caused by road construction and use.

5. Excavator and bulldozer operators and forest road inspectors and supervisors should be trained in and informed about environmentally-friendly procedures.

## Competing interests

The authors declare that they have no competing interests.

## Authors’ contribution

Author read and approved the final manuscript.
